# Immunogenicity and safety of a second dose of a measles-mumps-rubella vaccine administered to healthy participants 7 years of age or older: A phase III, randomized study

**DOI:** 10.1080/21645515.2018.1489186

**Published:** 2018-07-12

**Authors:** Remon Abu-Elyazeed, William Jennings, Randall Severance, Michael Noss, Adrian Caplanusi, Michael Povey, Ouzama Henry

**Affiliations:** a GSK, Philadelphia, PA, USA; b Radiant Research, San Antonio, TX, USA; c Radiant Research, Chandler, AZ, USA; d Radiant Research, Cincinnati, OH, USA; e GSK, Wavre, Belgium; f GSK, Rockville, MD, USA

**Keywords:** immunization schedule, immunogenicity, measles-mumps-rubella vaccine, safety, second dose

## Abstract

The introduction of vaccination programs against measles, mumps, and rubella (MMR) led to significant global reduction in morbidity and mortality from these diseases. The currently recommended MMR vaccination schedule in the United States of America comprises 2 vaccine doses typically administered at 12–15 months and 4–6 years, respectively. Considering recent outbreaks in the USA, catch-up vaccination with an additional dose of MMR vaccine could contribute to outbreak control and community protection. This phase III, observer-blind, randomized controlled trial (NCT02058563) assessed the immunogenicity and safety of a dose of the MMR-RIT vaccine (*Priorix*, GSK) compared to MMR II vaccine (control; *M-M-R II*, Merck&Co Inc.) in ≥7-year-olds who had received ≥1 previous dose of MMR vaccine. We assessed anti-measles, anti-mumps, and anti-rubella antibody geometric mean concentrations (GMCs; primary endpoint) and seroresponse rates (SRRs) at day 42 post-vaccination. Solicited, unsolicited, and serious adverse events (AEs) were recorded. The according-to-protocol cohort for immunogenicity included 869 participants (MMR-RIT: N = 433; MMR II: N = 436). We observed anti-measles, anti-mumps, and anti-rubella antibody GMCs of 1790.2 mIU/mL, 113.5 EU/mL, and 76.1 IU/mL, respectively, and SRRs of 98.8%, 98.4%, and 99.5%, respectively, after a dose of MMR-RIT; non-inferiority compared to MMR II was demonstrated. Both vaccines showed comparable reactogenicity profiles; the most common solicited AEs were injection site redness and pain, and fever (MMR-RIT: 12.2%, 11.8%, and 3.0%; MMR II: 11.7%, 11.5%, and 5.2%, respectively). The dose of MMR-RIT induced robust immune responses that were not inferior to those of MMR II, and was well tolerated.

## Introduction

Measles, mumps, and rubella diseases are highly contagious, common during childhood in the pre-vaccination era, and can lead to severe complications at different ages.^1^^-^^6^ To prevent these diseases, the World Health Organization (WHO) and the Centers for Disease Control and Prevention (CDC) currently recommend maintaining high immunization levels through universal routine vaccination of all children with 2 doses of a combined measles, mumps, and rubella (MMR) vaccine.^1^^,^^2^^,^^7^^,^^8^ The introduction of vaccination programs led to significant global reductions in morbidity and mortality from these diseases.^1^^,^^9^^,^^10^

In the United States of America (USA), 2 doses of the only licensed MMR vaccine, MMR II (*M-M-R II*, Merck & Co Inc.), are recommended for use in children ≥12 months old as the standard of care. This vaccination strategy eliminated endemic measles in the USA by the year 2000^11^ and endemic rubella by 2004;^5^ mumps cases have decreased >99% since the pre-vaccine era.^12^ However, measles and mumps outbreaks have recently arisen in the USA.^12^^,^^13^ Since measles was eliminated, the greatest number of measles outbreak cases in the USA occurred in 2014: 667 cases from 27 states. Measles virus can continue entering the country by infected travelers, and it can spread among communities with low vaccination coverage. Most measles cases occur in unvaccinated individuals.^13^^-^^16^ Mumps outbreaks, by contrast, can occur among highly vaccinated communities, in individuals who live in densely populated settings (e.g., college campuses and religious communities). Large outbreaks of mumps occurred in the USA in 2006 and 2016 (>6,000 cases each year), 2009 to 2010 (>3,500 cases), and 2017 (>5,500 cases up to September).^12^ To reduce the probability of outbreaks, completion of the MMR vaccination schedule is crucial. The Advisory Committee on Immunization Practices currently recommends administration of a first dose of MMR II at 12–15 months of age and a second dose typically at 4–6 years of age (however, the second dose can be administered as early as 28 days after the first dose).^8^ The second dose is intended to increase the percentage of seroprotected individuals in the vaccinated population, as some individuals are not protected after the first dose, thus contributing to a higher community protection. Administration of a dose at > 6 years of age might be needed in children, adolescents and adults who missed the second dose, in those who may lack documentation of prior 2-dose administration, in susceptible individuals in high-risk groups (e.g., college students, healthcare workers, military personnel), in immigrants or travelers without proper vaccination, and in outbreak settings.^8^ However, very scarce data are available on the protection provided by a dose of MMR vaccines when administered later than recommended for the second dose. To our knowledge, only 2 studies have been published that evaluated safety and/or immunogenicity of a second dose of MMR vaccine in individuals aged 11–13 years: one using only the combined MMR vaccine MMR II,^17^ and the other one comparing MMR II with the combined MMR vaccine MMR-RIT (*Priorix*, GSK).^18^

**Figure 1.**
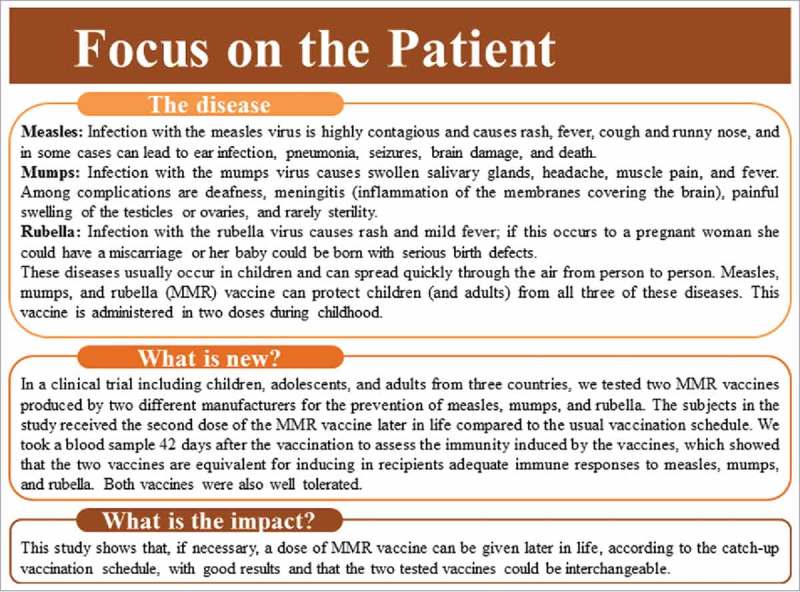
Focus on the Patient section.

We conducted a phase III, randomized study to investigate the immunogenicity and safety of a second dose of MMR-RIT when administered to individuals aged 7 years or older. The MMR-RIT vaccine is currently licensed outside the USA in over 100 countries, and it is recommended for use in individuals aged ≥9 months according to a 1- or 2-dose schedule depending on the country. The availability of another licensed MMR vaccine in the USA would mitigate the health risk of potential interruptions of the MMR II vaccine supply. Investigating the immunogenicity and safety of a MMR-RIT dose given later in life would provide data to support its use, when needed, and thus maintain the high levels of vaccination coverage recommended by the WHO and CDC. A summary contextualizing the outcomes of this study is displayed in the Focus on the Patient Section (Fig. 1) for the convenience of health care professionals.

## Results:

### Study participants

**Figure 2.**
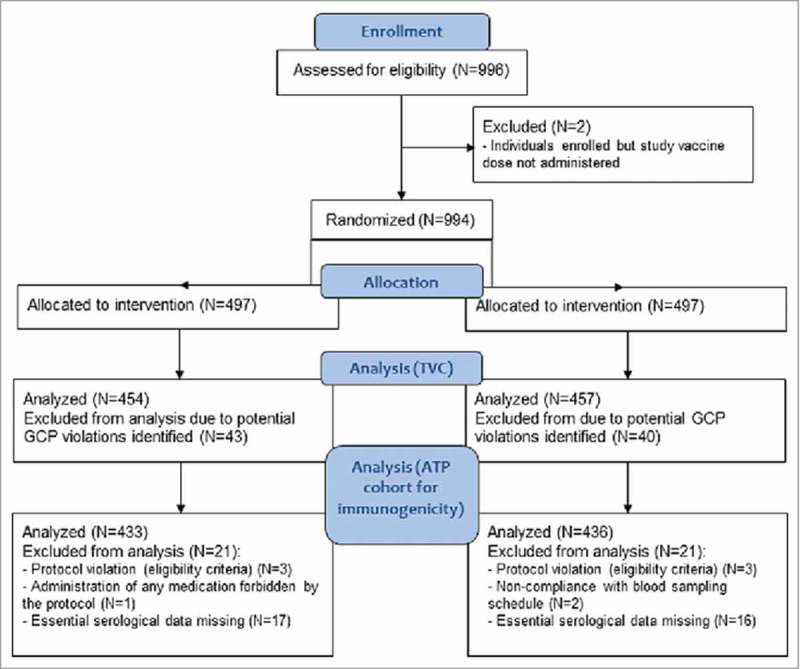
Flow diagram of the study participants. Footnote: N, number of participants; n, number of participants within the category; GCP, good clinical practice; TVC, total vaccinated cohort; ATP, according-to-protocol.

Of the 996 enrolled participants, 994 received 1 vaccine dose: 497 received the MMR-RIT vaccine (MMR-RIT group) and 497 received the MMR II vaccine (MMR II group) (Fig. 2). Forty-three participants in the MMR-RIT group and 40 in the MMR II group were excluded from the analyses due to significant Good Clinical Practice (GCP) concerns associated with 2 sites. As a result, the total vaccinated cohort (TVC) consisted of 911 participants (MMR-RIT: N = 454, MMR II: N = 457), of whom 95.4% were included in the according-to-protocol (ATP) cohort for immunogenicity (MMR-RIT: N = 433; MMR II: N = 436) (Fig. 2).

The demographic characteristics were similar and well balanced between the groups (Table 1); the mean age of participants was 25.7 years (standard deviation [SD] = 13.8 years). Almost two-thirds (64.1%) of the participants were ≥18 years old; as per inclusion criteria, these participants were allowed to have received more than 1 previous dose of a MMR vaccine.

**Table 1.** Demographic characteristics of the study participants (total vaccinated cohort, N = 911).

Characteristic	MMR-RIT (N = 454)	MMR II (N = 457)
Age(years), mean (SD)	25.9 (13.9)	25.6 (13.8)
Age category, n (%)		
<18 years	162 (35.7)	165 (36.1)
≥18 years	292 (64.3)	292 (63.9)
Females:males	250:204	252:205
Geographic ancestry, n (%)		
White—Caucasian/European heritage	334 (73.6)	344 (75.3)
African heritage/African American	108 (23.8)	103 (22.5)
Other	12 (2.6)	10 (2.2)

N, number of participants; SD, standard deviation; n (%), number (percentage) of participants in the category.

* Age at study vaccination.

### Immunogenicity assessments

At Day (D) 42 post-vaccination, we observed an antibody geometric mean concentration (GMC) of 1790.2 milli international units (mIU)/mL for anti-measles, 113.5 enzyme-linked immunosorbent assay (ELISA) units (EU)/mL for anti-mumps, and 76.1 IU/mL for anti-rubella antibodies in the MMR-RIT group (Table 2). The primary objective of non-inferiority for MMR-RIT vaccine over MMR II in terms of anti-measles, anti-mumps, and anti-rubella antibody concentrations was demonstrated, as the lower limits of the 95% confidence intervals (CIs) of the adjusted GMC ratios (MMR-RIT over MMR II) at D42 were ≥0.67 for all 3 antibodies (Table 2).

**Table 2.** Non-inferiority of MMR-RIT vaccine compared to MMR II in terms of anti-measles, anti-mumps and anti-rubella adjusted geometric mean antibody concentrations at Day 42 (ATP cohort for immunogenicity).

	Adjusted GMC		
Antibody	MMR-RIT (N = 432)	MMR II (N = 435)	Adjusted GMC ratio (MMR-RIT GMC/MMR II GMC) Ratio (95% CI)
Anti-measles (mIU/mL)	1790.2	1781.5	1.00 (**0.91**, 1.11)
Anti-mumps (EU/mL)	113.5	107.8	1.05 (**0.96**, 1.16)
Anti-rubella (IU/mL)	76.1	74.6	1.02 (**0.93**, 1.11)

ATP, according-to-protocol; GMC, geometric mean concentration; N, number of participants with both pre- and post-vaccination results available; CI, confidence interval.

a The two-sided 95% CI for the adjusted GMC ratio was obtained using an ANCOVA model on the logarithm-transformed concentrations including the vaccine group as fixed effect, gender, age and country groups as continuous effects, and the pre-vaccination log-transformed concentration as regressor.

Bolded values indicate lower limit of the two-sided 95% confidence interval ≥0.67 (i.e., criterion for non-inferiority of MMR-RIT over MMR II in terms of GMCs).

At D42, MMR-RIT vaccination yielded a seroresponse rate (SRR; defined as an immunoglobulin G [IgG] antibody concentration ≥200 mIU/mL for anti-measles, ≥10 EU/mL for anti-mumps, and ≥10 IU/mL for anti-rubella irrespective of the baseline antibody concentrations) of 98.8% for anti-measles, 98.4% for anti-mumps, and 99.5% for anti-rubella antibodies. The secondary objective of non-inferiority for MMR-RIT vaccine over MMR II vaccine in terms of anti-measles, anti-mumps, and anti-rubella SRRs was met, as the lower limits of the 95% CIs of the differences in SRRs (MMR-RIT minus MMR II) at D42 were ≥-5% for all 3 antibodies (Table 3).

**Table 3.** Non-inferiority of MMR-RIT vaccine compared to MMR II in terms of anti-measles, anti-mumps and anti-rubella seroresponse rates at Day 42 (ATP cohort for immunogenicity).

	SRR (%)		
Antibody (prespecified threshold)	MMR-RIT (N = 433)	MMR II (N = 436)	Difference in SRRs (MMR-RIT SRR – MMR II SRR) % (95% CI)
Anti-measles (≥200 mIU/mL)	98.8	99.1	-0.24 (**-1.87**, 1.32)
Anti-mumps (≥10 EU/mL)	98.4	99.5	-1.16 (**-2.90**, 0.23)
Anti-rubella (≥10 IU/mL)	99.5	99.8	-0.23 (**-1.46**, 0.86)

ATP, according-to-protocol; SRR, seroresponse rate: percentage of participants with concentration equal to or above the prespecified threshold indicated for each assay; N, number of participants with available results; CI, confidence interval.

a The standardized asymptotic 95% confidence interval was obtained using the Miettinen and Nurminen method. Bolded values indicate lower limit of the standardized asymptotic 95% confidence interval ≥-5% (i.e., criterion for non-inferiority of MMR-RIT over MMR II in terms of SRRs).

We also conducted additional, exploratory subgroup analyses where we assessed the immune responses per age subgroup (<18 years and ≥18 years). A dose of either MMR-RIT or MMR II elicited similar immune responses in terms of adjusted GMC ratios and SRR differences regardless of the age group (Supplementary Tables 1 and 2).

**Figure 3.**
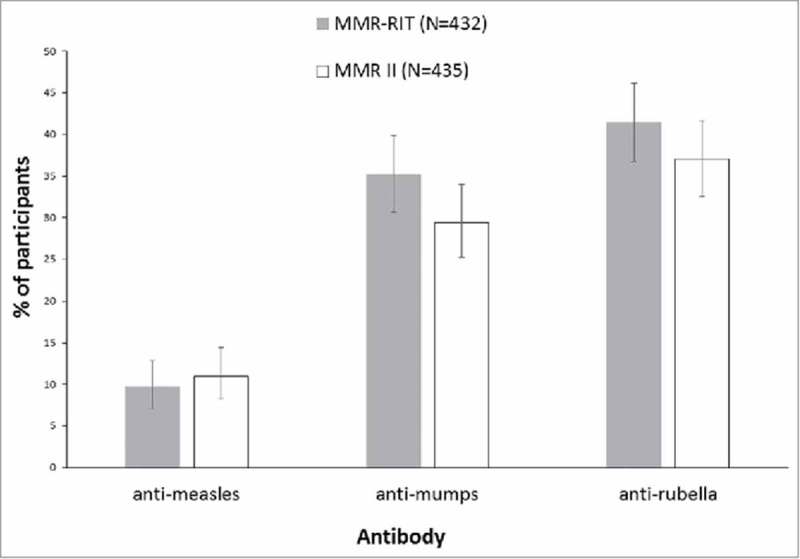
Percentage of participants who achieved a 4-fold or greater increase in anti-measles, anti-mumps, or anti-rubella virus antibody concentrations at Day 42 (ATP cohort for immunogenicity). Footnote: N, number of participants with both pre- and post-vaccination available results; ATP, according-to-protocol. For participants with a seronegative status at pre-vaccination, a 4-fold rise in antibody concentration is defined as 4 times the cut-off level of the assay. Cut-off levels for anti-measles, anti-mumps and anti-rubella virus antibody concentrations are 150 mIU/mL, 5 EU/mL and 4 IU/mL, respectively. The error bars represent the upper and lower limits of the two-sided 95% confidence intervals obtained using the Clopper Pearson method.

In this study, we included an immunogenicity analysis to investigate how many vaccinees would show a ≥4-fold increase in the concentration of anti-measles, anti-mumps, or anti-rubella antibodies after a single dose of the vaccine. At D42 after vaccination, we found that a similar percentage of participants in both vaccine groups showed a ≥4-fold increase in anti-measles (MMR-RIT: 9.7%; MMR II: 11.0%), anti-mumps (MMR-RIT: 35.2%; MMR II: 29.4%), and anti-rubella antibodies (MMR-RIT: 41.4%; MMR II: 37.0%) (Fig. 3). In both groups, more participants achieved a ≥4-fold increase in anti-rubella antibodies than in anti-mumps or anti-measles antibodies (Fig. 3).

### Reactogenicity and safety

**Figure 4.**
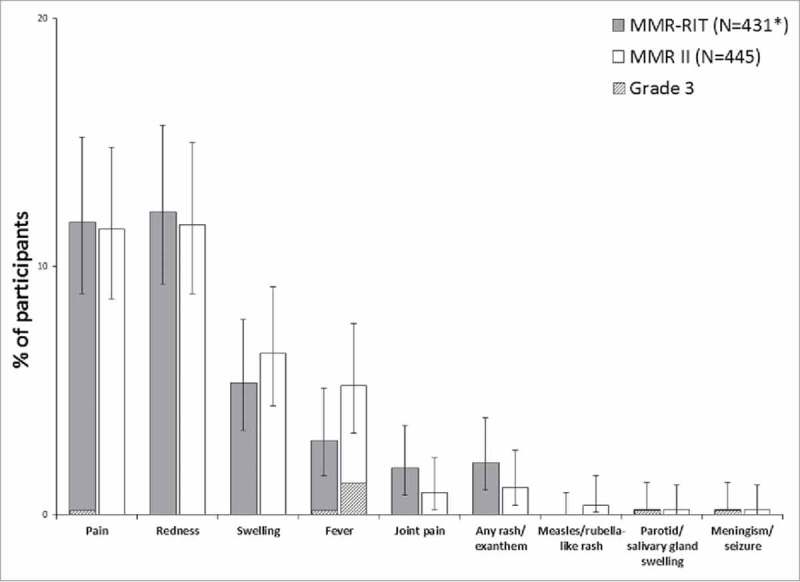
Incidence of solicited injection site (Day 0–3) and general adverse events (Day 0–42) (total vaccinated cohort). Footnote: N, number of participants with the documented dose with local symptoms sheets completed *Except for pain, redness, and swelling, for which MMR-RIT (N = 433). Fever: temperature ≥38°C. Grade 3 was defined as: limb was painful at rest, which prevented normal everyday activities (pain); diameter >50 mm (redness and swelling); temperature >39.5°C (fever); adverse event preventing normal, everyday activities (joint pain, rash/exanthem, meningism/seizure); swelling with accompanying general symptoms (parotid/salivary gland swelling). The error bars represent the upper and lower limits of the two-sided 95% confidence intervals obtained using the Clopper Pearson method.

The reactogenicity profile was similar between the 2 vaccine groups, in terms of both incidence and severity of the solicited adverse events (AEs) reported (Fig. 4). The most common solicited local AEs were redness (12.2% in MMR-RIT; 11.7% in MMR II) and pain (11.8% in MMR-RIT; 11.5% in MMR II). The most common solicited general AE was fever (3.0% in MMR-RIT; 5.2% in MMR II), followed by rash of any type (2.1% in MMR-RIT; 1.1% in MMR II) and joint pain (1.9% in MMR-RIT; 0.9% in MMR II). Cases of measles/rubella-like rash were very low in this study (0.0% in MMR-RIT; 0.4% [2 participants] in MMR II). The incidence of grade 3 solicited AEs was low in general, and similar between groups (Fig. 4).

We found a similar incidence of unsolicited AEs and serious AEs (SAEs) between groups (Table 4). Unsolicited AEs were reported by 20.9% (MMR-RIT) and 17.9% (MMR II) of the participants; the percentages of these AEs that were considered grade 3 or related to the study vaccine were similar between groups (Table 4). SAEs were reported by 0.7% (MMR-RIT) and 1.5% (MMR II) of the participants; none of them were related to the study vaccination and no fatal SAEs were reported in this study.

**Table 4.** Percentage of participants with unsolicited adverse events (Day 0–42) and serious adverse events (Day 0–180) (total vaccinated cohort).

	MMR-RIT (N = 454)		MMR II (N = 457)	
	n	% (95% CI)	n	% (95% CI)
Unsolicited AEs (≥1 AE)	95	20.9 (17.3, 25.0)	82	17.9 (14.5, 21.8)
Grade 3 (≥1 AE)	7	1.5 (0.6, 3.2)	5	1.1 (0.4, 2.5)
Related (≥1 AE)	12	2.6 (1.4, 4.6)	15	3.3 (1.8, 5.4)
SAEs (any, ≥1 SAE)	3	0.7 (0.1, 1.9)	7	1.5 (0.6, 3.1)

AE, adverse event; SAE, serious adverse event; N, number of participants with the administered dose; n/%, number/percentage of participants reporting a symptom at least once; CI, two-sided 95% confidence interval obtained using the Clopper Pearson method.

a AEs of grade 3 intensity were those preventing normal, everyday activities.

b Related AEs were considered by the investigator to be related or possibly related to the study vaccine.

## Discussion:

In this study, we assessed the immunogenicity and safety of a dose of the MMR-RIT vaccine when administered later than the routine recommended schedule of 4–6 years of age for a second dose, in comparison to MMR II (the only currently licensed vaccine in the USA). The immune responses observed following a MMR-RIT dose when administered to participants 7 years of age or older were robust and non-inferior to those obtained after a MMR II dose. This held true for anti-measles, anti-mumps, and anti-rubella GMCs and SRRs. The safety profiles of the 2 vaccines were similar; the most common solicited AEs were redness and pain at the injection site, and fever.

The safety and immunogenicity of a second dose of MMR-RIT vaccine administered at the recommended schedule of 2–6 years of age has already been described. In a previous phase III trial conducted in France, Germany and Italy, a second dose of MMR-RIT administered to 2–6-year-old children elicited robust immune responses, and ≥99% of the children aged 2–6 years were seropositive after vaccination.^19^ In our study, when we administered a dose of the same vaccine at ≥7 years of age, we also obtained robust immune responses for all 3 antibodies despite the age difference.

There are scarce data in the literature on the immunogenicity and safety of MMR vaccines administered outside of the recommended schedule. In a phase III study conducted in Sweden, the immune response of a second dose of either MMR-RIT or MMR II was compared in 12-year-olds who had been primed with MMR II at 2 years of age.^18^ In that study, for most of the participants (who were initially seropositive), MMR-RIT elicited equal or stronger immune responses than MMR II except for GMTs for anti-measles.^18^ In our study, we found similar immune responses between MMR-RIT and MMR II groups as measured in terms of adjusted GMCs, SRRs, and percentage of participants with a ≥4-fold antibody increase after vaccination; however, we did not take into account the pre-vaccination serostatus of the participants.

Another study, published in 1996, compared the immune responses to a second dose of MMR II vaccine when given at either 4–6 years or 11–13 years of age.^17^ After the second dose, 100% of the vaccinees in both age groups were seropositive for all 3 antigens. In our study, the SRRs after a dose of MMR vaccine administered at ≥7 years of age were ≥98.4% for all 3 antigens, and MMR-RIT SRRs were non-inferior to MMR II SRRs.

The results of these 2 previous studies outside the routine recommended schedule together with those of our study indicate that a dose of a MMR vaccine, including MMR-RIT, seems to induce robust immune responses even when it is administered later than the routine recommended schedule of 4–6 years of age.

In our study, the reactogenicity profile was similar between the vaccine groups in terms of both incidence and severity of AEs; both vaccines were well tolerated by the study participants and no safety concerns were raised. In the present study, we specifically assessed joint pain as young adults, especially women, are susceptible to arthralgia or arthritis upon mumps infection.^20^ We did not observe any differences between vaccine groups in the incidence of joint pain.

In the previous phase III trial conducted in France, Germany and Italy, where the immunogenicity and safety of a second dose of MMR-RIT at 2–6 years of age were evaluated, the most common solicited local AEs were redness and pain. Fever was the most frequently reported solicited general AE.^19^ Redness, pain, and fever were also the most common solicited AEs in our study. In both studies, the incidence of rashes was comparable and low (rash of any type: ≤3.4%; measles/rubella-like rash: ≤0.4%).

In the previous Swedish study that assessed immunogenicity and safety of a second dose of the MMR-RIT and MMR II vaccines administered outside of the recommended schedule, redness, pain, and swelling were the most frequent solicited local AEs and fever was the most frequent solicited general AE.^18^ As in our study, the incidence of all these AEs was similar between groups in the Swedish study except for pain, which was more frequent in the MMR II group (33.3%) than in the MMR-RIT group (20.1%).^18^

In light of the results from our study and previous studies, we can conclude that a dose of MMR-RIT administered later than 6 years is well tolerated, as is the second dose of MMR-RIT administered at the recommended schedule of 4–6 years of age, with fever, redness, and pain being the most common solicited AEs.

The present study has some limitations. In our study, it was difficult to recruit participants younger than 18 years because most of them had already received more than 1 dose of a MMR-containing vaccine (exclusion criterion of this study). Consequently, although we enrolled individuals older than 7 years, most of the participants (64.1%) were adults aged 18 years or older (mean age 25.7 years). Our age subgroup analyses, however, showed that the non-inferiority of MMR-RIT compared to MMR II held true when subjects younger than 18 years and subjects 18 years or older were analyzed separately. To our knowledge, this is the first study reporting the immune responses of adults to an additional dose of MMR-RIT vaccine. This may be relevant for older susceptible individuals at high risk, such as military personnel, healthcare workers, travellers, and immigrants.

In our study, participants aged 18 years or older may have received 2 doses of MMR vaccine prior to enrolment as they were allowed to self-report having 1 or more prior doses of MMR vaccine. In fact, our records show that approximately 5% of the enrolled participants confirmed the receipt of 2 prior doses—although this percentage may be an underestimation because some adults may have self-reported 1 instead of 2 prior doses. In adults with 2 prior MMR doses, the dose we administered (intended as a second dose) was actually a third dose. Even though that could have introduced some variability in the results obtained, we allowed >1 prior dose and self-report in adults to optimize participant recruitment, as many adults do not have access to their childhood vaccination records. Of note, although it is difficult to know the exact number of adults with 2 prior doses enrolled in this study, it is very unlikely that all adults received 2 prior doses because at the time these adults were children the recommendation was to receive only 1 dose.

Our study population was heterogeneous in terms of time since first dose of MMR vaccine. Moreover, although the mean age of participants was similar between groups (MMR-RIT: 25.9 years; MMR II: 25.6 years), the span of ages included was considerably broad (7–59 years) and the SDs of these mean ages were larger than expected (MMR-RIT: 13.9 years; MMR II: 13.8 years). Nevertheless, our GMCs and SRRs results (i.e., the statistically powered endpoints) are robust because we took into consideration the greater SDs in the statistical design of this study.

The results of this study suggest that administration of a MMR vaccine dose later in life in individuals who may have not received a second dose at the currently recommended schedule would increase the vaccination coverage and community protection. This could decrease the probability of measles outbreaks, as most of the outbreak cases occur in settings with a higher proportion of unvaccinated or undervaccinated individuals. Moreover, it has been recently suggested that an additional (i.e., third) dose of MMR vaccine could be beneficial to reduce the number of cases during a mumps outbreak.^21^ As noted earlier in the discussion, in our study, few adults (5%) had received a third dose of MMR vaccine due to the inclusion criterion requiring them to have at least—but not exactly—1 prior dose of MMR vaccine. Our age sub-group analyses showed non-inferiority of MMR-RIT in the population 18 years or older, which are, in our experience, the group most likely to seek immunization in outbreak situations.

Altogether, our results support the notion that a dose of MMR-RIT could be given later than the routine recommended schedule of 4–6 years, if needed, without being inferior to a dose of the only currently licensed vaccine in the USA, MMR II. Provided that it is granted regulatory approval in the USA, MMR-RIT could be an efficacious alternative for catch-up immunization of individuals 7 years of age or older for preventing measles, mumps and rubella. In addition, MMR-RIT could be administered safely and effectively in people primed with a different measles-containing vaccine.

## Patients and methods

### Study design and participants

We conducted a phase IIIA, observer-blind, randomized, controlled, multicenter study (NCT02058563) between July 2014 and September 2015 in 3 countries: the USA, Estonia, and Slovakia.

**Figure 5.**
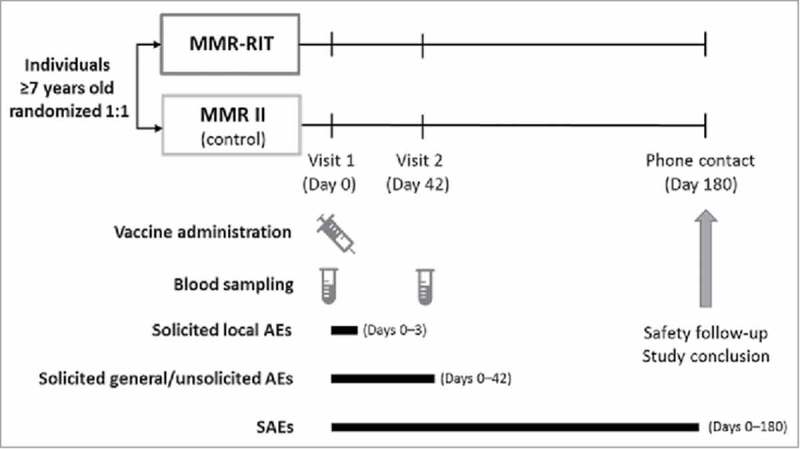
Study design. Footnote: AEs, adverse events; SAEs, serious adverse events.

We randomized healthy participants aged ≥7 years in a 1:1 ratio to receive 1 dose of either MMR-RIT vaccine or MMR II vaccine (Fig. 5). The randomization was performed using a central system, with treatment numbers allocated by dose and with a minimization procedure accounting for gender, age, and country. Data were collected in an observer-blind manner: the vaccinees, the laboratory in charge of the laboratory testing, and those responsible for evaluating any study point were blinded to the treatment.

The study consisted of 2 site visits (D0 and D42) and 1 phone contact for safety follow-up (D180). All participants received 1 dose of the assigned vaccine at D0. Blood samples were collected at D0 and D42.

Participants complying with the requirements of the protocol who were born after 1956 and were in stable health (as determined by investigator's assessment of medical history and physical examination), with a previous administration of a MMR-containing vaccine (written documentation was required for participants <18 years old; written or verbal documentation was allowed for older participants), with no history of measles, mumps, or rubella disease and for whom written informed consent was obtained (from the participants or their parent(s)/legally acceptable representative) were eligible for the study. Female participants of childbearing potential were enrolled only if they used adequate contraception and had a negative pregnancy test on the day of vaccination.

Children in care and children 7–17 years of age who received more than 1 dose of a MMR-containing vaccine, as well as individuals who received any MMR-containing vaccine within 42 days before the study vaccination, were ineligible for the study. Other exclusion criteria are detailed in the supplementary material.

An independent ethics review committee or an institutional review board approved the study protocol, which is available at https://www.gsk-clinicalstudyregister.com/study/115231. The study was conducted in accordance with the Declaration of Helsinki and the GCP guidelines. All mandatory laboratory health and safety procedures were complied with during the conduct of this study. External organizations were contracted to help conduct the research (study monitoring and laboratory assays).

### Study objectives

The primary objective of this study was to demonstrate the non-inferiority of the MMR-RIT candidate vaccine versus the MMR II vaccine (only currently licensed vaccine in the USA) in terms of antibody concentrations (anti-measles, anti-mumps, and anti-rubella) at D42. The secondary objectives were: 1) to demonstrate the non-inferiority of the MMR-RIT candidate vaccine versus the MMR II vaccine in terms of SRRs to measles, mumps, and rubella viruses, 2) to assess the percentage of participants who achieved ≥4-fold increase in antibody concentrations after vaccination, and 3) to assess the safety and reactogenicity of both vaccines.

### Study vaccines

The MMR-RIT vaccine contains live attenuated measles virus (Schwarz strain) ≥10^3.0^ cell culture infectious dose 50 (CCID_50_), mumps virus (RIT4385 strain) ≥10^4.3^ CCID_50_, rubella virus (Wistar RA 27/3 strain) ≥10^3.0^ CCID_50_, anhydrous lactose, sorbitol, mannitol, amino acids, and neomycin. The MMR II vaccine contains live attenuated measles virus (Moraten Edmonston-Enders strain) ≥10^3.0^ tissue culture infectious dose 50 (TCID_50_), mumps virus (Jeryl Lynn strain) ≥10^4.1^ TCID_50_, rubella virus (Wistar RA 27/3 strain) ≥10^3.0^ TCID_50_, sorbitol, sodium phosphate, sucrose, sodium chloride, hydrolyzed gelatin, human albumin, fetal bovine serum, and neomycin. Each participant received 1 dose of the assigned vaccine by subcutaneous injection in the upper left arm.

### Immunogenicity assessments

We assessed the antibody GMCs against each virus at D42 as the primary endpoint of the study. The secondary immunogenicity endpoints were: 1) the SRR at D42 (SRR was defined as an IgG antibody concentration ≥200 mIU/mL for anti-measles, ≥10 EU/mL for anti-mumps, and ≥10 IU/mL for anti-rubella irrespective of the baseline antibody concentrations; these thresholds were accepted by the USA Food and Drug Administration as offering clinical benefit); and 2) the percentage of participants with a ≥4-fold increase between D0 and D42 in GMCs for each antigen.

We determined the concentration of IgG antibodies in the blood samples taken at D0 and D42. We used commercial ELISA kits for anti-measles, anti-rubella (Enzygnost, Dade Behring), and anti-mumps antibodies (Pharmaceutical Product Development, Inc).

### Reactogenicity and safety assessments

We assessed the reactogenicity and safety of the vaccines as secondary endpoints of the study. Solicited local AEs (injection site pain, redness, and swelling) were recorded from D0 to D3; solicited general AEs were recorded from D0 to D42 (Fig. 5), and included: fever (defined as temperature ≥38°C), rash (both measles/rubella-like and any rash), swelling of the parotid or other salivary glands, meningism, and joint pain (arthralgia or arthritis). Unsolicited AEs were recorded from D0 to D42, whereas serious AEs were recorded throughout the entire study period (D0 to D180).

We graded solicited AEs according to their intensity (grade 1–3), with grade 3 defined as: limb was painful at rest, which prevented normal everyday activities (pain); redness or swelling of diameter >50 mm; temperature >39.5°C (fever); AE preventing normal, everyday activities (rash, meningism, joint pain); swelling with accompanying general symptoms (parotid/salivary gland swelling); AEs preventing normal, everyday activities (unsolicited AEs). All solicited local (injection site) reactions were considered causally related to vaccination. Causality of all other AEs was assessed by the investigator.

### Statistical analyses

We planned to enroll 1000 individuals to have the 800 evaluable participants expected to meet the primary endpoint. The power to simultaneously meet the primary objective and the secondary objective of non-inferiority in terms of SRRs was ≥92.7%. Due to the variable time since first vaccination with a previous MMR vaccine, the population enrolled in this study could be relatively heterogeneous, with GMC SDs higher than expected. Therefore, we used a conservative estimate of 0.60 as the reference SD for each antigen.

The TVC included all vaccinated participants from sites with no significant GCP concerns. The ATP cohort for immunogenicity included participants who received the vaccine as per protocol and who had post-vaccination immunogenicity results for at least 1 of the 3 antigens.

Statistical analyses were performed using the Statistical Analysis Systems (SAS) version 9.2 on Windows and StatXact-8.1 procedure for SAS. For the prespecified immunogenicity analyses, the adjusted GMCs and the adjusted GMC ratios (MMR-RIT GMC over MMR II GMC) for each antigen were tabulated with their 95% CI. The non-inferiority of MMR-RIT over MMR II in terms of GMCs would be demonstrated if the lower limit of the 2-sided 95% CI of the adjusted GMC ratio at D42 was ≥0.67 for all the antigens tested. The SRRs and the difference in SRRs between groups (MMR-RIT SRR minus MMR II SRR) for each antigen were tabulated with their 95% CI. The non-inferiority of MMR-RIT over MMR II in terms of SRRs would be demonstrated if the lower limit of the standardized asymptotic 95% CI of the difference in SRRs at D42 was ≥-5% for all the antigens tested.

All the secondary objectives were analyzed descriptively except the assessment of non-inferiority in terms of SRRs (described above). Descriptive analyses of GMCs and SRRs by age subgroup (<18 years and ≥18 years) were also performed.

## Trademark statement

*Priorix* is a trade mark owned by or licensed to the GSK group of companies. *M-M-R II* is a trademark of Merck and Co., Inc. *Enzygnost* is a trademark of Dade Behring Marburg GmbH.
